# Efficient 3D conformer generation of cyclic peptides formed by a disulfide bond

**DOI:** 10.1186/s13321-022-00605-8

**Published:** 2022-05-03

**Authors:** Huanyu Tao, Qilong Wu, Xuejun Zhao, Peicong Lin, Sheng-You Huang

**Affiliations:** School of Physics, Huazhong University of Science and Technology, Wuhan, 430074 Hubei People’s Republic of China

**Keywords:** Cyclic peptide, Disulfide bond, Conformer generation, Peptide modeling, Peptide docking

## Abstract

Cyclic peptides formed by disulfide bonds have been one large group of common drug candidates in drug development. Structural information of a peptide is essential to understand its interaction with its target. However, due to the high flexibility of peptides, it is difficult to sample the near-native conformations of a peptide. Here, we have developed an extended version of our MODPEP approach, named MODPEP2.0, to fast generate the conformations of cyclic peptides formed by a disulfide bond. MODPEP2.0 builds the three-dimensional (3D) structures of a cyclic peptide from scratch by assembling amino acids one by one onto the cyclic fragment based on the constructed rotamer and cyclic backbone libraries. Being tested on a data set of 193 diverse cyclic peptides, MODPEP2.0 obtained a considerable advantage in both accuracy and computational efficiency, compared with other sampling algorithms including PEP-FOLD, ETKDG, and modified ETKDG (mETKDG). MODPEP2.0 achieved a high sampling accuracy with an average C$\alpha$ RMSD of 2.20 Å and 1.66 Å when 10 and 100 conformations were considered, respectively, compared with 3.41 Å and 2.62 Å for PEP-FOLD, 3.44 Å and 3.16 Å for ETKDG, 3.09 Å and 2.72 Å for mETKDG. MODPEP2.0 also reproduced experimental peptide structures for 81.35% of the test cases when an ensemble of 100 conformations were considered, compared with 54.95%, 37.50% and 50.00% for PEP-FOLD, ETKDG, and mETKDG. MODPEP2.0 is computationally efficient and can generate 100 peptide conformations in one second. MODPEP2.0 will be useful in sampling cyclic peptide structures and modeling related protein-peptide interactions, facilitating the development of cyclic peptide drugs.

**Supplementary Information:**

The online version contains supplementary material available at 10.1186/s13321-022-00605-8.

## Introduction

In recent years, peptides as potential drugs have been continuously explored [1]. Compared with small molecules and antibodies, peptides have unique advantages, such as strong selectivity and high binding affinity with protein targets [2]. However, as the most common peptides, linear peptides may have limitations like low bioavailability and poor half-life in circulation [3, 4]. A common strategy to overcome such limitations is to cyclize the peptide [5, 6]. Most cyclic peptides are closed by connecting head to tail or with a disulfide bond. Compared with linear peptides, cyclic peptides are more stable and have higher binding affinity, higher specificity, and improved enzyme activity, making cyclic peptides desirable drug candidates [7]. So far, more than 40 cyclic peptides have been approved by the FDA and EMA for clinical treatment [8, 9].

Structural information of peptides is important for modeling protein-peptide interactions and thus the design of cyclic peptide drugs in drug development [10–21]. However, peptides are highly flexible and exist as an ensemble of conformations in solution. Therefore, compared with the conformer generation of small molecules [22–25], determining the conformations of peptides is much more challenging [26]. Some computational peptide structure prediction methods have been developed to model cyclic peptides, such as PEP-FOLD [27–29], Peplook [30], and PEPstrMOD [31]. These methods can generate a certain number of peptide conformations, which can be used for docking [13–15, 32]. However, the prediction accuracy of these algorithms is still not satisfactory. Sampling cyclic peptides by molecular dynamics (MD) simulations is another way. Especially, it is a powerful strategy to study the structure of a certain cyclic peptide through MD simulations with enhanced sampling, such as Replica Exchange Molecular Dynamics (REMD) [33–35], Metadynamics [36, 37], and Bias-Exchange Metadynamics [38]. However, sampling cyclic peptide conformations through MD simulations is time-consuming and also difficult for non-experts.

Recently, we developed a fast de novo peptide modeling algorithm named MODPEP [39] to sample the three-dimensional (3D) conformers of linear peptides. By taking advantage of the one-letter (or one-amino acid), two-letter (or two-amino acid), and helical conformations in the experimentally determined peptide structures [40], MODPEP achieved an excellent performance in both accuracy and speed and can generate several hundred reasonable peptide conformers within seconds [39]. Inspired by the therapeutic potential of cyclic peptides, here we propose an extended version of MODPEP, named MODPEP2.0, to efficiently model the 3D structures of peptides cyclized through a disulfide bond. Our MODPEP2.0 algorithm first generates the cyclic part of the peptide based on a cyclic backbone library. Then, it builds the 3D structure of non-cyclic parts by adding amino acids one by one onto the cyclic fragment based on the constructed rotamer libraries. Our method was extensively tested on a dataset containing 193 cyclic peptides with one disulfide bond, and compared with three other approaches, including PEP-FOLD, ETKDG [41], and modified ETKDG (mETKDG) [42].

**Table 1:** The average accuracy of MODPEP2.0 measured by C$\alpha$ (cRMSD), backbone (bRMSD), and all heavy atoms (aRMSD) for the peptides with different length ranges when an ensemble of 100 conformations were considered for each peptide

Cyclic Peptide		RMSD (Å)		
Length	Number	cRMSD (Å)	bRMSD (Å)	aRMSD (Å)
(0, 10]	35	0.70	0.73	1.77
(10, 15]	83	1.28	1.24	2.59
(15, 20]	37	2.34	2.22	3.65
(20, 25]	32	2.64	2.52	4.00
(25, 30]	6	3.22	3.05	4.52
ALL	193	1.66	1.60	2.94

**Table 2:** The average accuracy and success rate by MODPEP2.0, PEP-FOLD, ETKDG, and mETKDG for the peptides with different length ranges when an ensemble of 100 conformations were considered for each peptide. The numbers in bold fonts indicate the best performances for the corresponding length ranges

Cyclic Peptide		cRMSD (Å)				Success Rate (%)			
Length	Number	MODPEP2.0	PEP-FOLD$^\mathrm{a}$	ETKDG$^\mathrm{b}$	mETKDG$^\mathrm{c}$	MODPEP2.0	PEP-FOLD	ETKDG	mETKDG
( 0, 10]	35	**0.70**	1.89	1.68	1.57	**100.00**	66.67	77.14	85.71
(10, 15]	83	**1.28**	2.19	2.70	2.44	**90.36**	65.06	38.55	56.10
(15, 20]	37	**2.34**	2.85	3.89	3.55	**64.86**	51.35	20.00	22.86
(20, 25]	32	**2.64**	3.65	5.98	4.69	**65.62**	28.12	0.00	0.00
(25, 30]	6	**3.22**	4.63	5.20	5.26	**33.33**	**33.33**	0.00	0.00
ALL	193	**1.66**	2.62	3.16	2.72	**81.35**	54.95	37.50	50.00

$^{\mathrm{a}}$The data of PEP-FOLD is based on the 182 cyclic peptides from 9 to 30 in length because PEP-FOLD cannot model 11 cyclic peptides with less than nine aa

${^{\mathrm{b}}}$The data of ETKDG is based on the 176 cyclic peptides modeled because ETKDG cannot model 17 cyclic peptides in the test set

${^{\mathrm{c}}}$The data of mETKDG is based on the 168 cyclic peptides modeled because mETKDG cannot model 25 cyclic peptides in the test set

## Materials and methods

MODPEP2.0 is an upgraded version of MODPEP, developed to perform conformational sampling of cyclic peptides formed by a disulfide bond. MODPEP2.0 samples cyclic peptides based on a cyclic backbone library. The non-cyclic residues are built from scratch one by one to the cyclic fragment using the similar strategy in MODPEP for modeling linear peptides. It should be noted that our method can be used to model any cyclic peptides, but has certain requirements on the amount of template data for the cyclic backbone fragments. Here, we restricted MODPEP2.0 to model the cyclic peptides formed by a disulfide bond due to the abundance of cyclic fragments with one disulfide bond in the PDB. As more and more experimental cyclic peptide structures are solved, it is expected that this method will be applicable to other cyclic peptides with more disulfide bonds.

### Cyclic backbone library construction

**Fig. 1:**
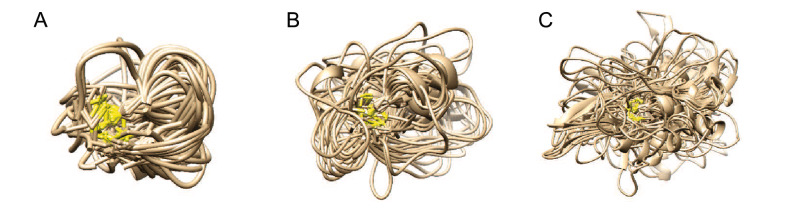
Examples of the cyclic backbone sub-libraries, where only the first 100 conformations are shown. The lengths of backbones are 6 (**A**), 12 (**B**), and 20 (**C**), respectively

We have constructed a length-dependent cyclic backbone library for cyclic peptides sampling. Specifically, we searched the protein structures in the PDB to get cyclic fragments meeting the following criteria. First, there is only one disulfide bond between the head and tail of the fragment and no more Cysteine residue among the fragment. Second, the length of the cyclic fragment is between 3 and 30. Third, there is no missing residue and backbone atom in the fragment. Here, we kept all atoms of Cysteine residues and only the backbone atoms of other residues (Fig. 1). This is because the side chain of Cysteine is the key component of disulfide bonds, and the side chain of other residues can be rebuilt from our single-letter library in MODPEP [39]. According to the lengths of cyclic fragments, we have a total of 28 sub-libraries corresponding to the lengths between 3 and 30. The structures in each sub-library were aligned according to their backbone atoms (C$\alpha$, N, C, and O), and clustered based on their backbone RMSDs (bRMSD). For different lengths of cyclic fragments, we chose different bRMSD cutoffs of $0.1\sqrt{n_\mathrm{cyc}}$ for clustering, where $n_\mathrm{cyc}$ is the length of the cyclic fragment. The bRMSD cutoff ranges from 0.17 Å for a fragment length of 3 and 0.55 Å for a fragment length of 30. Therefore, longer fragments have a larger bRMSD cutoff, which can evade the defect of bRMSD because longer fragments usually lead to larger RMSD values [43], to a certain extent. For each cluster with a certain length, we selected the cluster centroid as the representative.

### Cyclic peptide modeling

With the cyclic backbone library, MODPEP2.0 can automatically model the three-dimensional structures of a cyclic peptide from a peptide sequence and the given disulfide bond information. According to the cyclic length, MODPEP2.0 first selects a backbone template from the corresponding sub-library based on the probabilities, which depend on the sequence similarity between the target peptide and the backbone template as well as the resolution of the backbone template as follows,

1 $$\begin{aligned} P_i=\exp (w _\text{s}\times \frac{s_{i}}{s_\text{max}}+w _\text{r}\times \frac{r_{i}}{r_\text{max}}) \end{aligned}$$

where $s_{i}$ is the sequence identity score based on blosum62 matrix of the target sequence and the *i*-th backbone template, and $r_{i}$ is the resolution of the original protein that the *i*-th backbone template comes from. Here, the division by the corresponding maximum value is to normalize different contributions. We have changed the residue name of nonstandard residue in the backbone template to the nearest standard residue if it is modified from a standard residue according to the annotations in the PDB file, so the corresponding identity score mentioned in the following is based on the modified backbone sequences. The weight coefficients *w* of different contributions are empirically determined. Since the sequence similarity is much more important than the resolution of the template in homology modeling, we have empirically set the weights $w_{s}$ and $w_{r}$ to 6.0 and $-1.0$ in this study, respectively. This will enable MODPEP2.0 to choose a backbone template with a better identity score and a better resolution. Then we normalized the probability so that the sum in a cluster is 1.0. After selecting a backbone, MODPEP2.0 builds the side chains of residues in the cyclic backbone by selecting a corresponding rotamer according to the target sequence from the single-letter library mentioned in the previous version [39]. The residues outside the cyclic fragment were also built by the same way as in the previous study for modeling linear peptides [39]. While modeling a peptide, we also check the conformational diversity by calculating the backbone RMSDs between the newly-built model and all previously modeled peptide structures. If any RMSD is less than 1.0 Å, the model will be discarded. The peptide conformation modeling process repeats until a specified number of models have been successfully constructed.

### Test set

To construct a non-redundant test dataset of cyclic peptides cyclized by a disulfide bond, we filtered the PDB entries with the following criteria. First, the lengths of peptides are between 3 and 30 residues. Second, the peptide contains only one disulfide bond and the cyclic fragment has more than two residues. Third, the peptide contains standard amino acids only and there is no missing atom for the backbone. As of November 13, 2020, the query yielded a total of 310 peptides. Then, we used CD-HIT [44] to cluster the sequences of these peptides. If two sequences are the same in a cluster, the structure with the better resolution was retained. This resulted in a total of 200 non-redundant peptides. To test the modeling ability of tested programs for cyclic peptides, the peptides with the cyclic length less than 1/3 of the entire length were excluded from the test set. The final test data set consists of 193 cyclic peptides of length between 5 and 30. The detailed list is shown in Additional file 1: Table S1. To reduce the impact from the templates, we have also removed the corresponding sequences of the 193 cyclic peptides from the cyclic backbone library during the evaluation.

A new non-standard residue data set was constructed to evaluate the performance of our MODPEP2.0 method. We filtered the PepBDB [45] (2020-03-18) with the criteria as follows. First, the peptide contains at least one non-standard amino acid. The backbone atoms of the non-standard amino acids should be the same as those of standard amino acids, which are N, CA, C, and O. Second, the peptide is cyclized through a disulfide bond and the sequence length of cyclic fragment exceeds 1/3 of the entire peptide sequence. We used CD-HIT to cluster the sequences of screened peptides with a sequence identity cutoff 80%, and kept the peptide with the better resolution in a cluster. The final non-standard test data set consists of 9 cyclic peptides with lengths between 10 and 31. The detailed list is shown in Additional file 1: Table S2.

### Evaluation criteria

We used the same evaluation criterion as that in MODPEP [39] to assess the performance of tested approaches. Namely, the quality of a structure was measured by the root mean square deviation (RMSD) between the predicted model and the experimental structure of a peptide. The RMSD between the C$\alpha$ atoms of the peptide (cRMSD) was used as the primary evaluation parameter. The RMSD of backbone heavy atoms (bRMSD) and the RMSD of all heavy atoms (aRMSD) are additional quality assessment parameters to check the capability of MODPEP2.0 in modeling backbones and side chains. For an ensemble of *N* conformations of a peptide, the accuracy of this ensemble was defined as the RMSD of the best conformation, i.e., the smallest RMSD value.

As for the criterion for successful predictions, we have used a size-dependent RMSD cutoff as follows [39],

2 $$\begin{aligned} \mathrm {rmsd}(n)=1.0\times [1+\ln (n/n_{0})] \end{aligned}$$

where *n* is the length of the peptide and $n_{0}$ was set to 3 [39]. A successful prediction for a peptide of *n* residues was defined as a modeled peptide conformation with an RMSD within the cutoff of rmsd(*n*). In addition, unlike a large protein that has a relatively stable native structure, a short-length peptide normally may not have a unique structure because of its high flexibility. One important goal of peptide conformation generation is to generate an ensemble of peptide conformers that contains protein-bound structures, so that the biologically active ones can be screened during ensemble docking. Therefore, the top-1 performance would be less relevant than the top-*n* performance for a peptide conformation generation method in terms of ensemble docking. As such, we have used the best RMSD in an ensemble of peptide conformations for each peptide to measure the performance of a peptide modeling approach in this study.

### Compared with other methods

Only a few approaches allow modeling cyclic peptides with user-specified sequences, so it is difficult to compare MODPEP2.0 with other methods. Here, we have selected a widely used de novo peptide structure prediction server PEP-FOLD [27] and a conformer generation algorithm RDKit [46, 47]. PEP-FOLD predicts the Structural Alphabet (SA) probability profile based on an SVM model and performs peptide 3D generation through an enhanced greedy procedure, followed with a Monte-Carlo refinement. Coarse-grained Cysteine interaction force field and all-atom minimization based on Gromacs facilitate the formation of disulfide bonds. RDKit is one of the best and the most widely-used conformer ensemble generators for small molecules, which contains different algorithms to generate conformers. We have used two different RDKit algorithms for extensive comparison and reference. One is ETKDG (Experimental-Torsion-Knowledge Distance Geometry), which is the default conformer generator in RDKit (version 2015.03) [41]. ETKDG is not specifically designed for cyclic peptides, but a general molecular conformer generator, which performs stochastic search and utilizes distance geometry with chemical knowledge and experimental torsional-angle preferences. In addition, a modified ETKDG has been developed in 2020 and incorporated into the 2020.03 release of RDKit, which specifically facilitates the sampling of macrocycles [42]. The modified ETKDG contains additional torsional-angle potentials for aliphatic cyclic bonds and uses elliptical geometry and customizable Coulombic interactions as heuristics to restrict the search space of macrocycles. The inclusion of customizable Coulombic interactions is computationally expensive. We only tested the combination of modified ETKDG with eccentricity constraints, which is referred to as mETKDG in this study. It should be noted that PEP-FOLD, ETKDG, and mETKDG are *ab initio* structure prediction methods, and MODPEP2.0 relies on cyclic backbone templates. Therefore, the present comparison is to provide a performance reference more than a comparison.

**Table 3:** The average accuracy and success rate of MODPEP2.0 with different sequence identity cutoffs between the modeled peptide and the cyclic backbone library when an ensemble of 100 conformations were considered for a peptide

	Sequence identity cutoff (%)						
	100	90	80	70	60	50	40
cRMSD (Å)	1.66	1.70	1.74	1.87	1.92	1.99	2.09
Success Rate (%)	81.35	79.79	79.79	78.76	76.68	74.35	74.32

## Results and discussion

MODPEP2.0 can model cyclic peptides with one disulfide bond efficiently. The capacity of MODPEP2.0 in predicting near-native cyclic peptide structures was evaluated on a test set of 193 cyclic peptides and a dataset of 9 cyclic peptides with non-standard amino acids mentioned above. For a fair evaluation, we have removed the corresponding sequence from the cyclic backbone library when modeling a tested peptide. For each peptide, we have generated up to 1000 conformations based on its sequence and the disulfide bond information.

**Fig. 2:**
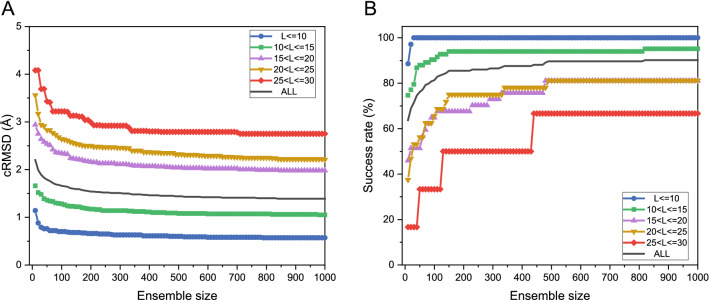
The average accuracy (**A**) and the average success rate (**B**) by MODPEP2.0 for peptides with different length ranges on the test set as a function of ensemble size. The detailed cRMSDs of predicted models for each peptide are listed in Additional file 1: Table S3

**Fig. 3:**
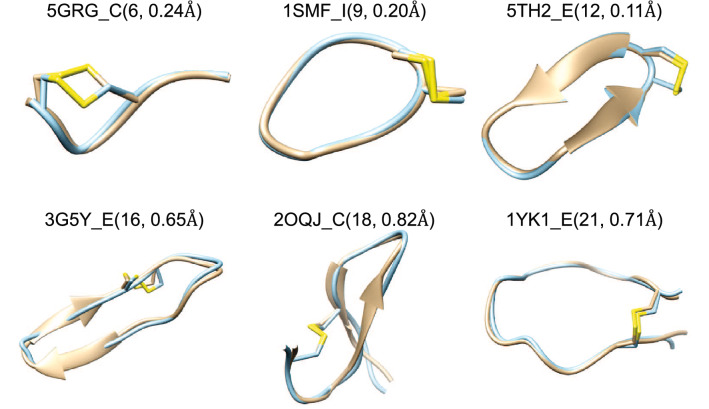
Six examples of successfully predicted models by for cyclic peptides when an ensemble of 100 conformations were considered. The predicted model (blue) is superimposed on the experimental structure (yellow). The corresponding peptide PDB code_chain ID is given above the structure with peptide length and corresponding accuracy in parentheses

### Accuracy

We modeled the 3D structures of 193 cyclic peptides in the test set and calculated the RMSDs of modeled peptide conformers. The test set was divided into five groups according to their sequence lengths, Fig. 2A shows the average accuracy as a function of ensemble size for the peptides of different length ranges. From the figure, we can find some notable features. First, MODPEP2.0 had a high prediction accuracy and a strong ability to sample cyclic peptides. The average accuracy for all peptides was below 2.0 Å for an ensemble of 100 conformations (Table 1). Second, it can be seen from Fig. 2 that the accuracy depends on the cyclic peptide length. The peptides within ten amino acids achieved an average cRMSD below 1.0 Å for an ensemble size of 100, indicating that MODPEP2.0 has an excellent performance on short cyclic peptide modeling. When the peptide lengths exceed 25 amino acids, the average cRMSD was about 3.5 Å for an ensemble of 100 conformations. The main reason for large RMSDs of long peptides is that the conformational space grows exponentially with the number of rotatable bonds. In addition, it is worth noting that there were only six cases ranging from 25 to 30 residues in length (Table 1), so the data for this group may lack sufficient statistics. Third, the accuracy also depends on the ensemble size of generated conformations. The accuracy changed quickly at small ensemble sizes and then became stable at big ensemble sizes. Overall, when an ensemble of 500 conformations was sampled, the accuracy was relatively stable. MODPEP2.0 can achieve an accuracy of 2.20 Å on average for an ensemble size of 10, 1.54 Å for an ensemble size of 200, 1.44 Å for an ensemble size of 500, and 1.39 Å for an ensemble size of 1000. Therefore, an ensemble of 500 conformations seems to be a good choice considering both accuracy and computational resource. However, other peptide modeling methods like PEP-FOLD can only provide up to 100 structures for download. Therefore, for a fair comparison, we have used 100 as the default ensemble size in the following evaluations. Of course, users can choose a larger size of samples according to their needs to achieve more accurate predictions.

To evaluate the robustness of MODPEP2.0, we also calculated the RMSD of the backbone atoms (bRMSD) and the RMSD of all heavy atoms (aRMSD) for an ensemble of 100 conformations. The average RMSDs of different length ranges are shown in Table 1. It can be seen from Table 1 that bRMSD and aRMSD showed the same trend as cRMSD, gradually increasing as the length increases. The bRMSD and cRMSD are also comparable, while aRMSD is larger. This suggests that cRMSDs can show the deviation of the overall backbone, but cannot include the information of side chains. The larger RMSD caused by the side chain can be understood by noting that we did not consider the environment of the peptide, including the solvents and proteins to bind. Different solvents and binding proteins will induce different side chain conformational changes. Therefore, it is difficult to predict the exact position of the side chain without considering the surroundings. For further research, users can use our predicted model as a starting point and consider the solvent and binding protein through molecular dynamic simulations or other methods.

**Fig. 4:**
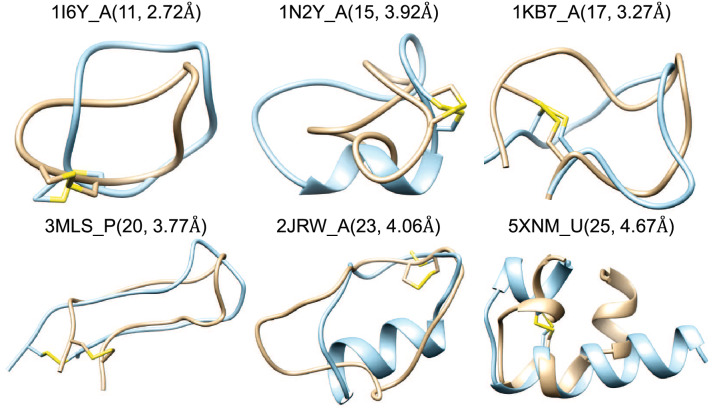
Six examples of failed models for cyclic peptides when an ensemble of 100 conformations were considered. The predicted model (blue) is superimposed on the experimental structure (yellow). The corresponding peptide PDB code_chain ID is given above the structure with peptide length and corresponding accuracy in parentheses

**Fig. 5:**
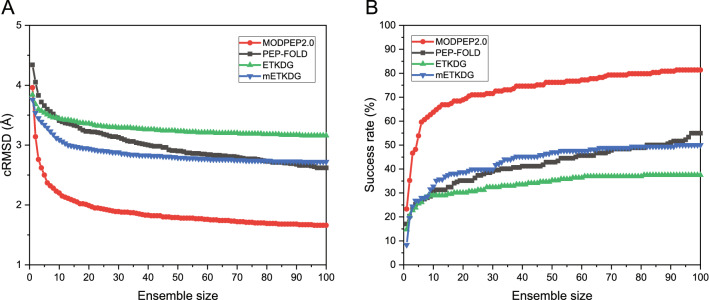
Comparison of the average accuracy (**A**) and the average success rate (**B**) between MODPEP2.0 and other methods (PEP-FOLD, ETKDG, mETKDG) as a function of ensembles size. The data of PEP-FOLD, ETKDG, and mETKDG are based on 182, 176, and 168 peptides that can be modeled, and MODPEP2.0 are based on the complete test set of 193 peptides

### Success rate

According to the RMSD cutoff defined in Eq. (1), we calculated the success rate of MODPEP2.0 in peptide conformer modeling. Here, the success rate is defined as the percentage of the peptides in the test set that includes at least one conformer within the corresponding RMSD cutoff for an ensemble of peptide conformations. The average success rates for different length ranges are listed in Table 2. It can be seen from the table that the success rate shows a similar trend to that of cRMSD. The average success rate for all peptides is 81.35% when an ensemble of 100 conformations were considered for a peptide. This indicates that MODPEP2.0 has a powerful cyclic peptide conformational sampling ability, and can reproduce near-native structures for most cyclic peptides. The sampling capacity of MODPEP2.0 depends on peptide lengths. For cyclic peptides with lengths ranging from 5 to 15, MODPEP2.0 achieved a success rate of more than 90% for an ensemble size of 100 conformations. For cyclic peptides with lengths ranging from 20 to 25, the average success rate decreased to 65.62% for an ensemble of 100 conformations. However, for cyclic peptides with lengths greater than 25, the average success rate is only 33.33% when an ensemble of 100 conformations are considered.

The success rate also depends on the ensemble size of sampled peptide conformations (Fig. 2B). The more conformations were generated, the higher the success rate was. On average, MODPEP2.0 gave a success rate of 63.73% for an ensemble size of 10, 85.49% for an ensemble size of 200, 89.64% for an ensemble size of 500, and 90.16% for an ensemble of 1000 conformations. As the ensemble size increases, the success rate grows rapidly at the beginning, and then gradually stabilizes. The success rate only increases slightly after more than 500 conformations. Therefore, MODPEP2.0 achieved a good balance between the ensemble size and the success rate when 500 conformations were considered. All groups showed a stable growth in the success rate except the last group of 25–30 residues, which exhibits a large rise at some points. The following reasons can explain this difference. First, there were only six peptide cases in the group of length ranging from 25 to 30. Therefore, even if one new test case succeeded, the success rate would show a great change. In addition, the length of the non-cyclic part is long for some cases, such as 5XNM_U shown in Fig. 4. The conformational space of the peptide grows exponentially with the length increases. As such, it is more challenging to model long peptides.

**Fig. 6:**
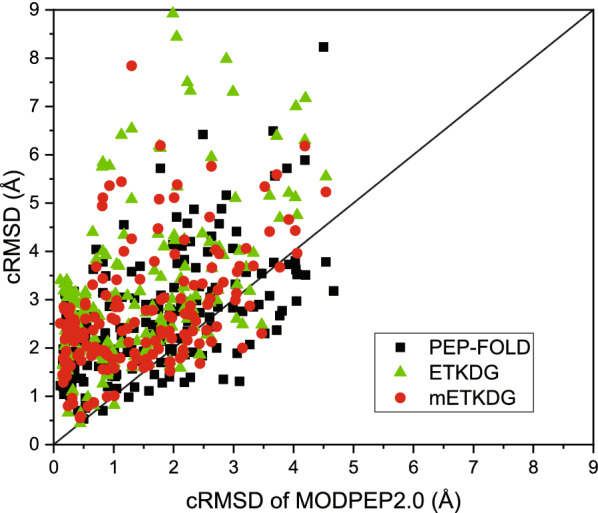
Comparison of the cRMSDs of predicted models between MODPEP2.0 and three other methods (PEP-FOLD, ETKDG, and mETKDG) on the test set of 193 cyclic peptides when an ensemble of 100 conformations were considered. The data of PEP-FOLD, ETKDG, and mETKDG are based on 182, 176, and 168 peptides that can be modeled

**Fig. 7:**
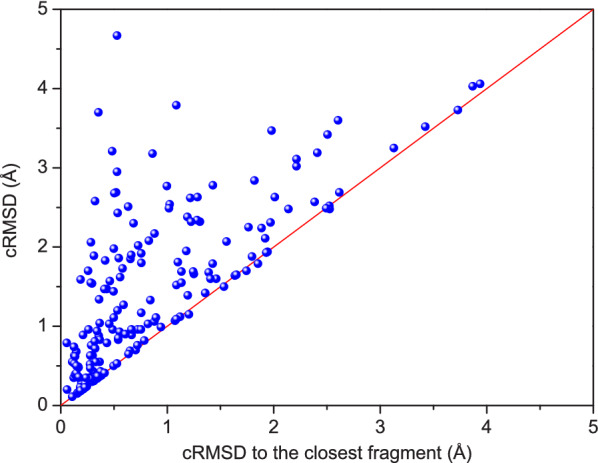
The best cRMSD of the modeled peptide structure with respect to the crystal conformation as a function of the similarity (cRMSD) to the closest cyclic fragment in the library on the test set of 193 cyclic peptides when an ensemble of 1000 conformations were considered. The red line stands for the function of $y=x$

### Examples of predicted models

Figure 3 shows several examples of peptide conformations that MODPEP2.0 successfully predicted. For these six examples, the accuracy was below 1.0 Å, resulting in high-quality predictions. These results suggested the accuracy of MODPEP2.0 in building cyclic peptide models. Nevertheless, MODPEP2.0 failed to sample correct conformations of some long cyclic peptides when an ensemble of 100 conformations were considered (Fig. 4). In addition to the large conformational space mentioned above, two additional factors may contribute to such failures. One is that there is no suitable cyclic backbone template in the library. 1N2Y_A, 3MLS_P, and 2JRW_A are the examples of this reason. It can be seen from the figure that the cyclic fragment of predicted models cannot overlap well with the experimental structure. The cRMSD of the closest cyclic backbone in the library to the experimental conformation is 3.73 Å for 1N2Y_A, 3.13 Å for 3MLS_P and 3.94 Å for 2JRW_A. Even if the best cyclic backbone was selected for modeling, the RMSD of the cyclic part would exceed the cutoff of a successful prediction, let alone considering the non-cyclic part of the peptide. For peptides that do not have a suitable backbone in the library, we can further try to model them through MD simulations or other methods. The other factor is that the probability formula in Eq. 1 could not select a good template when an ensemble of 100 conformations were considered. For example, the smallest cRMSD of 1I6Y_A in the cyclic backbone library is 1.94 Å  but the identity score between this template and the sequence of 1I6Y_A is only 18%, which was much smaller than the maximum score of 54% in the library. According to Eq. 1, the probability rank of this template is 241, making it difficult to pick this template when considering an ensemble size of 100 conformations.

**Fig. 8:**
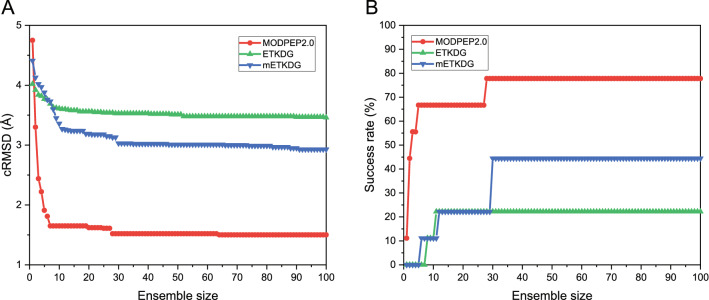
Comparison of the average accuracy (**A**) and the average success rate (**B**) between MODPEP2.0 and two RDKit methods (RDKit and mETKDG) as a function of ensembles size on a test set of 9 cyclic peptides with non-standard residues

### Comparative evaluations

We compared our MODPEP2.0 with the other three approaches mentioned above, including PEP-FOLD, ETKDG, and mETKDG. For PEP-FOLD, we submitted the different peptide sequences with the disulfide bond information in the test set to its online server and downloaded the modeled results. During the submission of PEP-FOLD jobs, all the default parameters were used except the “Type of simulation” where long simulations correspond to 200 runs were chosen for more extensive sampling. The PEP-FOLD server only allows downloading up to 100 clustered peptide models. For a fair comparison, we used all 100 peptide models with their cluster rank based on sOPEP energies. As PEP-FOLD can only process the disulfide bonded cyclic peptides with 9–36 amino acids, we could not have the PEP-FOLD results for 11 test cases with less than 9 amino acids, yielding a subset of 182 cyclic peptides in the evaluation of PEP-FOLD. For RDKit, we downloaded the program of version 2015.03 from its official website and ran it locally using the default parameters. For each peptide, we generated 100 conformations for each tested peptide by RDKit. ETKDG could not model 17 peptides, resulting in a total of 176 peptides in the evaluation. For mETKDG, we downloaded the program of version 2020.03 from its official website and ran it locally using the same parameters with eccentricity constraints in ref [42]. The total number of conformers generated is always divisible by 18, and we generated 108 conformers for each tested peptide. We only analyzed the results for the top 100 conformers. mETKDG could not model 25 peptides, yielding a final set of 168 peptides. Therefore, the accuracy and the success rate for PEP-FOLD, ETKDG, and mETKDG were evaluated on 182, 176, and 168 peptides, respectively.

Figure 5 shows the average accuracy and the average success rate of different methods as a function of ensemble size on the test set. It can be seen from the figure that MODPEP2.0 achieved the best accuracy and the highest success rate among the four methods, followed by PEP-FOLD and ETKDG. When the ensembles of 10, 50, and 100 conformations were considered, MODPEP2.0 obtained an accuracy of 2.20, 1.79, and 1.66 Å, compared with 3.41, 2.90, and 2.62 Å for PEP-FOLD, 3.44, 3.23, and 3.16 Å for ETKDG, 3.09, 2.79, and 2.72 Å for mETKDG, respectively. In addition, MODPEP2.0 achieved an average success rate of 81.35% when an ensemble of 100 peptide conformations were considered, compared with 54.95% for PEP-FOLD, 37.50% for ETKDG, and 50.00% for mETKDG.

We further compared the modeling capabilities of different methods for peptides with different length ranges when an ensemble of 100 conformations were considered (Table 2). The data in bold indicates the best performance for the corresponding length range. It can be seen from the table that MODPEP2.0 achieved the best performance for peptides of all length ranges. As the peptide length increased, all methods had different degrees of decreases in accuracy and success rate, as expected. The peptides with 10–15 residues had the most number of 83. In this length range, MODPEP2.0 achieved an average cRMSD of 1.28 Å, compared with 2.19 Å for PEP-FOLD, 2.70 Å for ETKDG, and 2.44 Å for mETKDG. Correspondingly, MODPEP2.0 had a high success rate of 90.36% for modeling peptides of this length range, which is considerably higher than 65.06% for PEP-FOLD, 38.55% for ETKDG, and 56.10% for mETKDG. For the six difficult targets whose lengths range from 25 to 30, although MODPEP2.0 and PEP-FOLD had the same success rate of 33.33%, the average accuracy of MODPEP2.0 was better than PEP-FOLD. Overall, MODPEP2.0 obtained an accuracy of 1.66 Å and a success rate of 81.35% in peptide conformer generation for the test set of 193 cyclic peptides.

In addition, the accuracy of the predicted conformations for each test case was also compared between MODPEP2.0 and the other methods (PEP-FOLD, ETKDG, and mETKDG). As shown in Fig. 6, MODPEP2.0 outperformed the other three methods on the benchmark and obtained a smaller cRMSD for most of the test cases than the other three methods. Specifically, MODPEP2.0 was better than PEP-FOLD for 142 cases, better than ETKDG for 161 cases, and better than mETKDG for 149 cases. Furthermore, it can be seen from Fig. 6 that there were many points distributed on the upper triangular region, suggesting the powerful ability of MODPEP2.0 in generating conformers of cyclic peptides with disulfide bonds.

### Impact of template similarity

We first explored the impact of sequence similarity between the modeled peptide and the cyclic backbone library. We removed the templates in the library whose sequence identity is greater or equal to a cutoff when modeling a peptide. A lower sequence identity cutoff corresponds to less similar sequences in the cyclic backbone library to the target. The cutoff of 100% returns to the results discussed above. Table 3 lists the average accuracy and success rate of MODPEP2.0 with different sequence identity cutoffs in the library. It can be seen from the table that the performance of MODPEP2.0 depends on the sequence similarity, gradually deteriorates as the sequence identity cutoff decreases. However, MODPEP2.0 still performed well at low sequence identity cutoffs. MODPEP2.0 achieved an average cRMSD of 1.74 Å at a sequence identity cutoff of 80%, 1.92 Å at a cutoff of 60%, and 2.09 Å at a cutoff of 40%, corresponding to success rates of 79.79%, 76.68%, and 74.32%, respectively. It is also encouraging to notice that MODPEP2.0 still performed significantly better than other methods mentioned above under different sequence identity cutoffs. This phenomenon may be attributed to both the low sequence-structure conservation for peptides and the structural diversity in the cyclic backbone library, which would facilitate the modeling of novel peptides.

Next, we examined the impact of the structural similarity between the modeled peptide and the cyclic backbone library. Figure 7 shows the cRMSD of the modeled peptide structure with respect to the crystal conformation as a function of the similarity (cRMSD) to the closest cyclic fragment in the library. It can be seen from the figure that the modeled peptides tend to have a better accuracy for a better template and most data points are located near the diagonal, which means that MODPEP2.0 is able to model comparably accurate structures to their closest cyclic fragments in the library for most of the test cases. However, there are a few cases like 5XNM_U and 3ZLD_B that have a significantly higher cRMSD (>3.0 Å) than the closest cyclic fragments in the library ($<0.5$Å). This can be understood because a peptide often contains both cyclic and non-cyclic parts, where the accuracy of cyclic parts tends to depend very much on the similarity to the existing fragments in the library, but the modeling of non-cyclic parts is more like a *de novo* way. For example, the peptides 5XNM_U and 3ZLD_B have 25 and 30 residues, but their cyclic parts only contain 10 and 12 residues, respectively (Additional file 1: Table S3). Encouragingly, despite their long lengths, these two peptides still achieved an accuracy of 4.67 Å and 3.7 Å, respectively. These results demonstrate the efficiency of our MODPEP2.0 utilizing the template fragments in the library.

### Performance on cyclic peptides with non-standard residues

We further tested MODPEP2.0 on a data set with non-standard residues. Figure 8 shows the average accuracy and success rate as a function of ensemble size. PEP-FOLD does not support modeling of non-standard amino acids, so there are no PEP-FOLD data on the non-standard residue test set. From the figure, we can observe similar notable features to those in Fig. 5. When the ensembles of 10, 50, and 100 conformations were considered, MODPEP2.0 obtained an accuracy of 1.65, 1.52, and 1.50 Å, compared with 3.61, 3.51, and 3.46 Å for ETKDG, 3.36, 3.01, and 2.93 Å for mETKDG, respectively. In addition, MODPEP2.0 achieved an average success rate of 77.78% when an ensemble of 100 peptide conformations were considered, compared with 22.22% for ETKDG, and 44.44% for mETKDG. Overall, MODPEP2.0 achieved roughly a similar prediction accuracy and success rate on the test sets of cyclic peptides with/without non-standard residues when an ensemble of 100 conformations were considered, indicating the robustness of MODPEP2.0.

## Conclusion

Cyclic peptides as drugs for various diseases have recently attracted a great attention. It is important to understand the relationship between the sequence and structure of peptides. Meeting the need, we have extended our MODPEP method to MODPEP2.0 to model the 3D structure of cyclic peptides formed by a disulfide bond. MODPEP2.0 samples cyclic peptide conformations based on the cyclic backbone library derived from the PDB. The non-cyclic residues are assembled from scratch one by one to the cyclic fragment. MODPEP2.0 is as fast as MODPEP and can generate 100 peptide conformations within one second. Being tested on a diverse benchmark of 193 cyclic peptides, MODPEP2.0 obtained an average accuracy of 1.66 Å and an average success rate of 81.35% in predicting experimentally observed peptide structures when an ensemble of 100 conformations were considered. As for cyclic peptides with non-standard residues, MODPEP2.0 achieved an average accuracy of 1.50 Å and an average success rate of 77.78% on a test set of 9 cyclic peptides with non-standard residues. MODPEP2.0 was also compared with three other methods, including PEP-FOLD, ETKDG, and mETKDG, and showed an overall best performance among these approaches. It is anticipated that MODPEP2.0 will facilitate the ensemble docking, help researchers discover new peptide therapeutics, and understand the sequence-structure relationship of cyclic peptides.

## Supplementary Information

**Additional file 1.** The PDB codes and chain ID for the two test sets and the detailed cRMSDs of the best model predicted by MODPEP2.0 on the test sets.

## Data Availability

The MODPEP2.0 program is available at http://huanglab.phys.hust.edu.cn/software/modpep2/.
